# Retraction: GMO debate: inconclusive

**DOI:** 10.3389/fgene.2013.00169

**Published:** 2013-08-20

**Authors:** Frontiers in Genetics Editorial Office

The journal wishes to retract the Opinion article cited above. Based on information reported after publication, this article was found to contain substantial sections that were taken verbatim from previous publications without proper quotation and citation.

The Journal and Chief Editor have decided to retract the article in its entirety and apologize to the readers of *Frontiers in Genetics*.

